# Supplementary material for: Policy effects of reduced pension contribution rate on innovation input——Empirical evidence from Zhejiang and Shandong provinces
Source: PLoS One. 2024 Dec 31;19(12):e0315841. doi: 10.1371/journal.pone.0315841 (PMC11687767; doi:10.1371/journal.pone.0315841)
Supplement: S1 Appendix — (DOCX) [file pone.0315841.s002.docx]

Appendix 1: Explanation of Synthetic Control Method

In assessing whether the reduction of pension insurance rates impacts enterprise innovation, researchers often prefer the difference-in-differences (DID) method to evaluate policy effects. However, the DID method requires that the treatment and control groups are comparable before policy implementation. This means that the classical DID approach depends on the assumption of a common trend. Given the significant variability in regional economic development across China and the use of macroeconomic data in this study, it is challenging to satisfy this condition, potentially leading to bias in the policy effect assessment. To address these limitations, the synthetic control method (Abadie & Gardeazabal, 2003) offers a more robust alternative for identifying policy effects. This method constructs a “counterfactual” reference group for each policy intervention by using the weighted average of the control group to create a synthetic control subject. In the context of this study, the method simulates the innovation levels of firms in a region where pension insurance rates have been reduced, as if the reduction policy had not been implemented. The effectiveness of the policy is then assessed by examining the discrepancy between the actual outcomes and the synthetic outcomes. Essentially, this approach represents a quasi-experimental study, where comparative experiments are conducted within the same region at the same point in time during the study period. The comparison between enterprise innovation in the region with and without the pension contribution rate reduction policy provides the basis for evaluating the policy’s impact on enterprise innovation.

Assume that there are N+1 regions and that region 1 reduces the employer pension contribution rate in period $T_{0}$, with no change in this rate in the other N regions. $Y_{1it}$ denotes the potential outcome of region i implementing a rate reduction in period t. $Y_{0it}$ indicates the potential outcome of region i not implementing a rate reduction in period t. The causal effect of implementing a rate reduction by region i is $\tau_{it}=Y_{1it}-Y_{0it}$, where *i*=1,···,*N*+1， and *t*=1,···,*T*. The outcome of enterprise innovation observed in region i in period t is $Y_{it}=D_{it}Y_{1it}+(1-D_{it})Y_{0it}=Y_{0it}+\tau_{it}D_{it}$, in which $D_{it}$ denotes whether region i is subject to the intervention of the rate reduction policy in period t. If yes, it takes the value of 1, otherwise 0. Assuming that region 1 is subject to the intervention of the rate reduction policy after period $T_{0}$ and other N regions are not subject to such intervention across all periods, the policy effect of the rate reduction can be expressed as $\tau_{1t}=Y_{11t}-Y_{01t}=Y_{1t}-Y_{01t}$. Since region 1 implements the rate reduction policy, the potential outcome observable in a period $t>T_{0}$ is $Y_{11t}$, while the potential outcome of the region when it is free of the policy intervention $Y_{01t}$ is unobservable. To estimate the counterfactual outcome of region 1, it can be represented by model (1) (Abadie et al., 2010):

$Y_{0it}=\delta_{t}+\theta_{t}Z_{i}+\lambda_{t}\mu_{i}+\varepsilon_{it}$ (1)

where $\delta_{t}$is the time-fixed effect; $Z_{i}$ is an observable (K×1)-dimensional covariate representing a control variable that is not affected by the rate reduction policy; $\theta_{t}$ is a (1×K)-dimensional unknown parameter vector; $\lambda_{t}$ is a (1×F)-dimensional unobservable common factor vector; $\mu_{i}$ is a (F×1)-dimensional coefficient vector; and $\varepsilon_{it}$ is the unobservable short-run shocks in each region, which is assumed to satisfy the mean value of zero at the regional level.

Model (1) is an extension of the classical DID model. The DID model allows for the existence of unobservable influence factors, but the effects of these factors do not vary over time. That is, $\lambda_{t}$ is a constant. However, in model (1), $\lambda_{t}$ is set to be a non-constant. In other words, the effects of unobservable factors can vary over time.

To derive $Y_{0it}$, it can be assumed that an (N×1)-dimensional weight vector $W=(w_{2}\cdots,w_{N+1})$ satisfies $w_{j}\geq0$, *j*=2,···,*N*+1, and $w_{2}+\cdots+w_{N+1}=1$. The weight is limited to be non-negative. This is equivalent to synthesizing the control group with a convex combination of the control group regions, thus avoiding possible bias due to extrapolation. Each particular value of vector W represents a synthetic control for region 1, which is a weighted average of all reference group regions. Weighting the variable values of each reference group region can be obtained:

$\sum_{j=2}^{N+1} w_{j}Y_{jt}=\delta_{t}+\theta_{t}\sum_{j=2}^{N+1} w_{j}Z_{j}+\lambda_{t}\sum_{j=2}^{N+1} w_{j}\mu_{j}+\sum_{j=2}^{N+1} w_{j}\varepsilon_{jt}$ (2)

Assuming that there exists a weight vector, it can be obtained that

$\sum_{j=2}^{N+1} w_{j}^{*}Y_{j1}=Y_{11}\text{，}\sum_{j=2}^{N+1} w_{j}^{*}Y_{j2}=Y_{12}\text{，}\cdots\text{，}\sum_{j=2}^{N+1} w_{j}^{*}Y_{jT_{0}}=Y_{1T_{0}}\text{，}\sum_{j=2}^{N+1} w_{j}^{*}Z_{j}=Z_{1}$ (3)

Abadie et al. (2010) proved that if $\sum_{t=1}^{T_{0}} \lambda_{t}^{'}\lambda_{t}$ is a non-singular matrix, it can be obtained that:

$Y_{01t}-\sum_{j=2}^{N+1} w_{j}^{*}Y_{kt}=\sum_{j=2}^{N+1} w_{j}^{*}\sum_{s=1}^{T_{0}} \lambda_{t}(\sum_{n=1}^{T_{0}} \lambda_{n}^{'}\lambda_{n})^{-1}\lambda_{s}^{'}(\varepsilon_{js}-\varepsilon_{1s})-\sum_{j=1}^{N+1} w_{j}^{*}(\varepsilon_{jt}-\varepsilon_{1t})$ (4)

It can be shown that under general conditions, equation (4) tends to 0. Thus, where $T_{0}<t<T$, the counterfactual outcome of region 1 can be approximately expressed as a synthetic control group, i.e., $\hat{Y}_{01t}=\sum_{j=2}^{N+1} w_{j}^{*}Y_{jt}$. On this basis, the policy effect can be estimated as follows:

$\hat{\tau}_{1t}=Y_{1t}-\sum_{j=2}^{N+1} w_{j}^{*}Y_{jt}\text{，}t\in[T_{0}+1\text{，}\cdots\text{，}T]$ (5)

The key to solving $\hat{\tau}_{1t}$ is to find the weight $W^{*}$ that make equation (3) hold, and $W^{*}$, the synthetic control vector, can be determined by getting an approximate solution. $W^{*}$ is determined by choosing to minimize the distance between $X_{1}$ and $X_{0}W$, that is $|X_{1}=X_{0}W|$. It is expressed as $|\text{|}X_{1}=X_{0}W||=\sqrt{(X_{1}-X_{0}W)^{'}V(X_{1}-X_{0}W)}$. $X_{1}$ is the (m×1)-dimensional eigenvector of a region before the pension contribution rate reduction. $X_{0}$ is an (m×N)-matrix with the jth column being the corresponding eigenvector before the rate reduction in region j. V is an (m×m)-symmetric semipositive definite matrix. Although the derivation procedure here is valid for any V, in practice the choice of V affects the estimated mean square error. In this paper, V is computed using the procedure developed by Abadie et al. (2010), which allows the synthetic region to approximate the innovation trajectories of firms in the region where rate reductions were made prior to the implementation of the policy. The enterprise innovation in the synthetic region obtained through the weighting operation actually simulates the enterprise innovation in a region subject to the rate reduction policy by assuming the absence of the policy. The difference in enterprise innovation between a region where the rate reduction policy is implemented and the synthetic region is the net effect of the pension contribution rate reduction policy on enterprise innovation in that region.
