# Supplementary material for: Policy effects of reduced pension contribution rate on innovation input——Empirical evidence from Zhejiang and Shandong provinces
Source: PLoS One. 2024 Dec 31;19(12):e0315841. doi: 10.1371/journal.pone.0315841 (PMC11687767; doi:10.1371/journal.pone.0315841)
Supplement: Attachment — Submitted filename: Response to Reviewers.docx [file pone.0315841.s003.docx]

Dear Editor and Reviewers,

Thank you for your suggestions, which are crucial for further optimization of my paper.

The revisions I have made are marked in blue in this paper.

The revised paper consists of the following parts:

1. A response letter to review comments, which has been uploaded as a separate file and labeled “Response to Reviewers”.

2. The revised paper, titled “Revised Manuscript with Track Changes”.

3. The revised paper without tracked changes, named “Manuscript”.

4. The project funds financing the research in this paper are added to the “Cover Letter”.

Revisions made as suggested:

1. The format of this paper has been adjusted and file of the paper has been named according to the requirements of the journal.

2. Relevant research data have been submitted upon request.

3. The responses to review comments are as follows:

Reviewer 1:

1. Reasons for choosing Zhejiang Province and Shandong Province as research targets.

The statutory pension contribution rate for urban workers in China is first coordinated at the municipal level and then at the provincial level. In practice, most provinces implement the statutory contribution rate of 20%, as mandated by the state. However, a few provinces have adopted different contribution rates due to their unique economic circumstances. These provinces include Guangdong, Fujian, Zhejiang, and Shandong. In Guangdong Province, pension contribution rates varied significantly across cities before the provincial statutory contribution rate of 14% was implemented. Similarly, Fujian Province set its pension contribution rate at 18% as early as 2002. Given these, neither province is suitable for inclusion in this study. In contrast, the pension contribution rates in Zhejiang Province were reduced to a range of 12% to 16% in 2009, while Shandong Province officially lowered its rate to 18% between 2010 and 2011. Prior to these reductions, the employer pension contribution rate in both provinces had been set at 20%. Thus, the lowered pension contribution rates in Zhejiang and Shandong Provinces provide a “quasi-natural experiment” to evaluate the economic effects of the pension contribution rate reduction policy on enterprise innovation.

2. References to explain why R&D intensity is used as a proxy for innovation investments.

A reference has been added to this paper, reflecting that it draws on Hall et al. (2008) to use R&D intensity as a proxy for innovation investments.

3. Research gaps between this paper and previous studies.

Previous research findings have been added to the introduction section along with the differences between the research in this paper and the previous research.

4. Theoretical insights

Theoretical insights are marked in blue in this paper.

Reviewer 2:

Thank you very much for your suggestions about the weaknesses of this paper. The following changes have been made accordingly.

1. Time frame: The selected time period of this paper is 2000-2017, which is mainly because, in 2019, the Chinese government reduced the pension contribution rate from 20% to 16%. At the end of the same year, the COVID-19 pandemic broke out, and over the subsequent three years, the central government temporarily exempted enterprises facing operational difficulties from pension contributions. As a result, this later period is not suitable for the analysis in this study. Therefore, the time frame used in this paper concludes in 2017, excluding the years that followed.

2. Regional selection: Why Zhejiang Province and Shandong Province are chosen as research targets

Following the implementation of the 20% pension contribution rate in China, only four provinces adopted a lower rate: Guangdong, Fujian, Zhejiang, and Shandong. In Guangdong, actual contribution rates varied significantly among different cities, making it less suitable for consistent analysis. Fujian lowered its statutory pension contribution rate to 18% in 2002, which is too distant from the relevant timeframe of this study. On the other hand, Zhejiang and Shandong announced pension contribution rate reductions in 2009 and 2010, respectively, after previously adhering to the national rate of 20%. This provides a “quasi-natural experiment” for assessing the effects of pension contribution reductions on enterprises’ innovation investments, making these two provinces ideal research targets.

3. Regional differences: Addressing the reviewer’s suggestion on industrial structure and enterprise size.

The reviewer’s suggestion to explore specific regional characteristics, such as industrial structure and enterprise size, is valuable. While this paper utilizes macroeconomic data, China’s statistical yearbooks do not provide detailed categorizations of these factors, making it challenging to conduct a more in-depth analysis with the available data. However, recognizing the importance of these dimensions, I intend to gather more comprehensive data in the future to conduct follow-up research.

4. Lack of mechanism analysis

The reviewer’s suggestion is indeed valuable. A detailed analysis of the causal mechanism behind how lowered pension contribution rates impact enterprises’ innovation investments is not fully explored in this paper, which represents a limitation. Existing research suggests that the effects of pension contribution rate reductions on innovation inputs can be examined from multiple perspectives, such as labor costs, financing costs, and tax incentives. However, this paper only briefly touches upon these mechanisms from a theoretical standpoint and lacks the micro-level data needed to further substantiate the conclusion. Since the paper relies on macroeconomic data rather than micro data from individual enterprises—such as employee wages, pension premiums, and financing costs—there is no detailed mechanism analysis included. In future research, I aim to gather micro-level data to provide a more thorough exploration of these mechanisms.

5. Sectoral analysis

The use of macro data limits the ability to conduct sectoral analysis in this paper. This is primarily due to the fact that sector-specific data in China’s provincial statistical yearbooks are neither comprehensive enough nor consistently available over time. Unlike micro data, which include a variety of enterprise-level elements, macro data do not offer the granularity necessary for analyzing different sectors effectively. To address this shortcoming, I plan to carry out further research in this field using micro-level data.

6. Theoretical framework

The theoretical framework of this paper has been extended to provide a more detailed discussion of relevant economic theories, particularly those that address the relationship between labor costs and innovation.

Key theories on labor costs and enterprise innovation have now been incorporated into the theory section.

7. Methodological complexity: Explanation of synthetic control method

An explanation of the synthetic control method has been added to the appendix.

8. Data presentation

The reviewer’s suggestion for clearer data presentation is important. Although this paper includes various graphs and charts, their clarity and intuitiveness could be improved to enhance the reader’s understanding.

The diagrams in this paper, except for Figure 1, are the output from STATA, which makes it challenging to create editable diagrams for inclusion in a Word document, as required by *PLOS ONE*. According to the journal’s formatting guidelines, diagrams must be submitted as editable figures, which could affect the quality of visualization, especially when figures like Fig. 2(a) and Fig. 2(b) are ideally displayed side by side to intuitively show the R&D expenditure gap between Zhejiang and synthetic Zhejiang. Presenting them in separate columns within a Word document might compromise the visual clarity. This explanation is provided to clarify the potential limitations in achieving optimal presentation according to the specific requirements.

9. References ordering

The references in this paper are arranged based on the order of citation. If required, I can adjust the references to alphabetical order based on the authors’ last names to comply with journal-specific requirements.
